# Commentary: “Consistent Superiority of Selective Serotonin Reuptake Inhibitors Over Placebo in Reducing Depressed Mood in Patients with Major Depression”

**DOI:** 10.3389/fpsyt.2015.00117

**Published:** 2015-08-21

**Authors:** Eiko I. Fried, Lynn Boschloo, Claudia D. van Borkulo, Robert A. Schoevers, Jan-Willem Romeijn, Marieke Wichers, Peter de Jonge, Randolph M. Nesse, Francis Tuerlinckx, Denny Borsboom

**Affiliations:** ^1^Faculty of Psychology, University of Leuven, Leuven, Belgium; ^2^Department of Psychiatry, Interdisciplinary Center Psychopathology and Emotion Regulation (ICPE), University Medical Center Groningen, University of Groningen, Groningen, Netherlands; ^3^Department of Psychology, University of Amsterdam, Amsterdam, Netherlands; ^4^Faculty of Philosophy, University of Groningen, Groningen, Netherlands; ^5^Department of Psychiatry and Psychology, School for Mental Health and Neuroscience, Maastricht University, Maastricht, Netherlands; ^6^School of Life Sciences, Arizona State University, Tempe, AZ, USA

**Keywords:** selective serotonin reuptake inhibitors, major depressive disorder, network analysis, antidepressants, symptomics

In the past decades, almost all research in psychiatry and clinical psychology has been directed at the level of *disorders*, such as major depressive disorder (MDD) or schizophrenia. As has been argued by many scholars in recent work, this organization of the psychiatric research program has yielded limited insights, which justifies the investigation of psychopathology at a more fine-grained level: the level of *symptoms* (1, 2). In the present letter, we indicate two primary directions for this research program, which we propose to call *symptomics*. We will focus our discussion on MDD specifically and discuss possibilities in relation to the recently published work by Hieronymus et al. (3).

Firstly, research has now shown that distinct depression symptoms, such as sad mood or insomnia differ in the risk factors that predispose them (4, 5), their underlying biology (6, 7), their response to specific life events (8, 9), and their impact on impairment of psychosocial functioning (10, 11) [for a review, see Ref. (1)]. This presents the first direction of the research agenda: to further investigate the properties in which individual symptoms differ from each other. The recently published work by Hieronymus et al. (3), “Consistent superiority of selective serotonin reuptake inhibitors over placebo in reducing depressed mood in patients with major depression”, adds the differential reactivity of depression symptoms to antidepressant medication to the prior body of work. In their analysis of clinical trial data of 6,669 patients with MDD published in *Molecular Psychiatry*, the authors document that depressive symptoms responded differentially to treatment with selective serotonin reuptake inhibitor (SSRI) antidepressants. Pooled effect sizes ranged from 0 (for symptoms, such as gastrointestinal and genital symptoms) to 0.44 (for depressed mood, a core symptom of depression). Hieronymus et al. (3) argue that these findings are consistent with prior antidepressant research that found differential treatment effects on symptoms and stress the importance of analyzing individual depression symptoms in future studies. We would like to extend their claim: these results, along with previous symptom-based findings, mandate the examination of *symptom-specific effects* throughout the realm of psychopathology.

The second research direction is the investigation of distinct patterns of *causes and effects* in which symptoms operate. Network analysis provides a tool to investigate these specific associations between symptoms that can sustain mental disorders (2). Contrasting the traditional explanation that the co-occurrence of symptoms (such as the depressive syndrome) is due to one underlying shared origin (MDD causes depression symptoms), networks conceptualize depression as a complex dynamic system of mutually reinforcing associations (12, 13). Figure 1 presents an example of such a psychopathological network – in the form of a Markov random field (MRF) – for the Hamilton Depression Rating Scale (HRSD), the same instrument analyzed by Hieronymus et al. (3). We computed the network from the enrollment symptom data of 3,467 patients from the antidepressant trial “sequenced treatment alternatives to relieve depression” (STAR*D) (14), a dataset that can be requested at the NIMH. The network can be viewed as a tentative estimate of the causal skeleton of a disorder and may be used to gauge which symptoms are most central in receiving input, and/or sending out influences into the system (15). Applying a network perspective to the paper by Hieronymus et al. (3) gains considerable analytic power. For example, the centrality of the STAR*D HRSD symptoms, as measured by their closeness to other symptoms in the network (2), correlates *r* = 0.53 (*p* < 0.05) with the symptom effect sizes reported by the authors. This means that more central symptoms exhibit greater reactivity to the intervention. In addition, symptoms with a higher closeness have a higher reported baseline severity (*r* = 0.60, *p* < 0.05) and symptoms with a higher baseline severity exhibit a (much) larger effect size (*r* = 0.77, *p* < 0.001). Thus, an interesting three-way pattern arises with more central HRSD items exhibiting higher reported means and higher reported reactivity to interventions.

**Figure 1:**
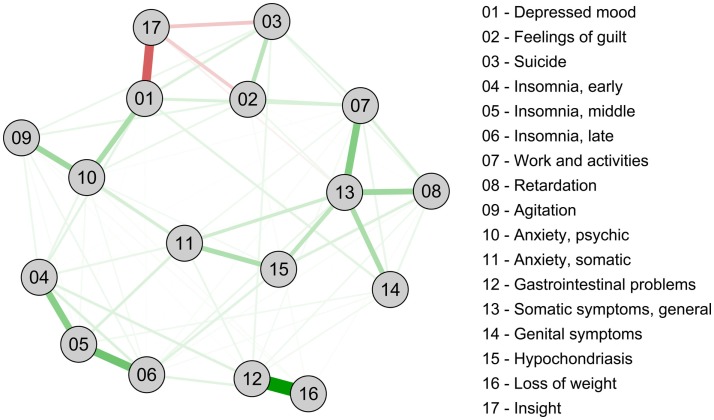
**Network of 17 HRSD depression symptoms**. Green lines represent positive associations, red lines negative associations, and thickness and brightness of an edge indicate the strength of the association.

While we can only speculate as to what produces this intriguing pattern of effects, the most important message is that focusing on the level of *symptoms* and analyzing the *causal relations* among them is likely to extend our understanding of psychopathology directly and significantly. The widespread reliance on disorders and the associated focus on symptom sum-scores in investigations of the biology and treatment of psychopathology may have concealed crucial insights (1, 16). A number of multivariate approaches have been developed for, and used with, depression symptoms previously, including structural equation models and network analyses (4, 9); in addition, time-series analysis studying network dynamics has become available as a tool to zoom in on the micro-level interactions among symptoms (17). Paying close attention to symptoms and their dynamics may have important clinical implications. Due to the highly heterogeneous nature of MDD (18, 19), individuals may differ substantially from each other not only in the symptoms they exhibit, but also in the way their symptoms are related to contextual influences, and in the way symptoms shape each other across time. A treatment focus on especially prevalent and central symptoms, instead of the categorically defined and heterogeneous disorders itself, may help increase the currently disappointing levels of treatment response (20). A broader investigation of symptom-specific treatment effects similar to the study performed by Hieronymus et al. (3) would enable clinical trials to match participants to specific treatments, based on their symptom profiles and dynamics.

In summary, symptomics invites the application of new modeling efforts to the level of individual symptoms as fundamental building blocks of mental disorders. As such, it may herald a time of renewed research energy that could, finally, provide an inroad to achieve real understanding of the mechanisms underlying psychopathology.

## Conflict of Interest Statement

The authors declare that the research was conducted in the absence of any commercial or financial relationships that could be construed as a potential conflict of interest.
